# Evaluating the Combined Effects of an Adverse Childhood Experiences–Focused Family Advocate Model and the Strengthening Families Program: Study Protocol for a Hybrid Type 1 Effectiveness-Implementation Trial in 36 New Jersey Communities

**DOI:** 10.2196/85151

**Published:** 2026-04-09

**Authors:** Dallas Elgin, Phillip W Graham, Kiersten Johnson, Sazid Khan, Michael Mills, Feker Wondimagegnehu, Brent Gibbons, Jason Williams, Xinyi Jiang, Andrea Zapcic, Brittany Simon

**Affiliations:** 1 RTI International, 3040 East Cornwallis Road, Durham, NC, 27713-2852, United States, 1 919248-8595; 2 Independent Scholar, Rockville, MD, United States; 3 National Center for Injury Prevention and Control, Centers for Disease Control and Prevention, Atlanta, GA, United States; 4 New Jersey Prevention Network, Tinton Falls, NJ, United States; 5 RWJBarnabas Health Institute for Prevention and Recovery, Eatontown, NJ, United States

**Keywords:** adverse childhood experiences, substance use, social determinants of health, clinical trial, implementation science, cost-effectiveness analysis

## Abstract

**Background:**

Early exposure to adverse childhood experiences (ACEs), such as parental substance use, elevates the risk of future substance use and drug overdose, and in the absence of intervention, could perpetuate a cycle of substance-related ACEs across generations. Although research suggests that effectively decreasing the prevalence and impact of ACEs and substance use can benefit from addressing both family- and community-level factors in tandem, there is a critical gap in the evidence base pertaining to interventions that effectively integrate these 2 factors to prevent substance use and ACEs.

**Objective:**

This study aims to conduct a rigorous evaluation of a novel intervention that integrates the established, evidence-based Strengthening Families Program with clinically trained, trauma-informed family advocates who will assist families in accessing resources related to ACEs and social determinants of health.

**Methods:**

This study uses a hybrid type 1 effectiveness-implementation trial design, which is used to test a clinical intervention while gathering information on its delivery during the effectiveness trial and on the potential for implementation in real-world settings. Our hybrid type 1 design consists of 3 components. A cluster-randomized controlled trial will be used to test the effectiveness of the intervention on substance use, overdose, and ACEs in 36 New Jersey communities. A robust process evaluation informed by the Consolidated Framework for Implementation Research will be used to explore implementation barriers and facilitators. A cost evaluation will be conducted to accurately estimate the costs required to implement the intervention components and assess the cost-effectiveness of the intervention.

**Results:**

Funding was awarded in 2022, and institutional review board approval was obtained in November 2023. Intervention and evaluation development lasted approximately 1 year, with study partners engaged in the co-design process to ensure alignment with the principles of community-placed behavioral health research. Study recruitment began in November 2023 and is anticipated to continue until September 2026. Final data collection is anticipated by March 2027, followed by data analysis.

**Conclusions:**

This study evaluates a novel intervention that integrates New Jersey’s established, evidence-based Strengthening Families Program with clinically trained, trauma-informed family advocates who will assist families in accessing community resources. By evaluating both family- and community-level outcomes through a hybrid type 1 design, the study bridges a key gap in the evidence base and provides a framework for combining multilevel interventions to reduce the long-term impacts of ACEs and substance use on youth and families. Future findings will provide critical insight related to intervention effectiveness, implementation barriers and facilitators, and the intervention’s associated costs and cost-effectiveness.

## Introduction

Adverse childhood experiences (ACEs) are preventable, potentially traumatic events that occur during childhood (0‐17 y), such as experiencing neglect, experiencing or witnessing violence, or having a family member attempt or die by suicide [1,2]. ACEs are common among US adults—in a survey conducted from 2011 to 2020, approximately two-thirds of respondents reported having had at least 1 ACE, and approximately 1 in 6 reported 4 or more ACEs [3]. Toxic stress resulting from ACEs can negatively affect healthy brain development, leading to increased risk for adverse mental and physical health outcomes, violence victimization and perpetration, and reduced economic and life opportunities [4-7]. Furthermore, ACEs can play a role in substance use initiation among youth [8,9]. Existing research indicates that ACEs are associated with an earlier age of initiating opioid use and injection drug use, developing substance use disorders [10,11], and experiencing lifetime overdose, all of which have played a significant role in fueling the opioid epidemic [8,12]. Given the acceleration of overdose deaths in the United States since at least 1999 [13], efforts to prevent risk factors for substance use initiation, such as ACEs, are important.

To improve public health across the lifespan, the US Centers for Disease Control and Prevention (CDC) invested resources in ACE prevention, spanning the focus areas of surveillance, research, and programmatic efforts [14]. In addition, the CDC’s National Center for Injury Prevention and Control (NCIPC) has synthesized the best available evidence on ACEs prevention into 6 comprehensive strategies to prevent ACEs and mitigate their long-term consequences if they do occur. One of these strategies is intervening to mitigate both immediate and long-term harms [2].

The Strengthening Families Program (SFP), a highly structured, evidence-based family skills training preventive intervention, has been recognized for its effectiveness in reducing known risk factors for ACEs and decreasing youth substance use, including opioid misuse [2,15-19]. The SFP7–17 curriculum is a new 11-session class curriculum from the developers of the original SFP [20]. Parents, youth, and children in SFP attend weekly 2-hour family group sessions for 11 weeks. A recent scoping review underscores the positive effects of SFP on substance use prevention and protective parenting factors in the United States [21]. Nevertheless, this scoping review highlighted a lack of available evidence regarding SFP adoption and maintenance at the setting level by the implementing organizations [21]. Indeed, SFP facilitators (“family coaches”) often lack clinical training to make effective referrals, limiting their ability to identify and address substance use–related issues or make service linkages in the community. Meanwhile, family-level interventions—such as patient navigators, family advocates (FAs), and family navigation programs—have been shown to increase protective factors against ACEs and reduce disparities while improving reach via service linkages in the community [22-25]. Research suggests that effectively decreasing the prevalence and impact of ACEs and substance use can benefit from addressing both family- and community-level factors in tandem [26,27]. However, little is known about the evidence base pertaining to interventions that effectively integrate family- and community-level factors to prevent or mitigate substance use and ACEs.

To address this critical gap in the evidence base, Research Triangle Institute (RTI) International and its partners, the New Jersey Prevention Network and the RWJBarnabas Health Institute for Prevention and Recovery, will evaluate a novel intervention that combines family- and community-level factors in 36 New Jersey communities experiencing a disproportionate burden of substance use and ACEs. This intervention will integrate clinically trained, trauma-informed FAs into New Jersey’s established evidence-based SFP. FAs will help families connect with community resources dedicated to the prevention and treatment of substance use and ACEs. This study will test whether integrating a combined family- and community-level intervention increases referrals and linkages to community resources, increases positive family functioning, and reduces youth and parent substance use and ACEs compared with families participating in SFP alone. The study will also test whether integrating a combined family- and community-level intervention produces system-level changes by aligning services and resources and subsequently decreasing the prevalence of substance use and ACEs at the community level. The purpose of this protocol paper is to provide an overview of an ongoing study by describing the study rationale, evaluation design, analysis methods, associated ethical requirements, and dissemination plan. Study findings will be presented in subsequent publications.

## Methods

### Research Objective and Aims

Our objective is to conduct a rigorous evaluation of a novel intervention approach that incorporates family- and community-level factors to mitigate the harms of exposure to ACEs and prevent future ACEs while aiming to prevent substance use and overdose. The study has three aims, that are, (1) use a cluster-randomized controlled trial (RCT) to test effectiveness on substance use, overdose, and ACEs of the SFP+FA intervention (treatment group) and SFP-only (control group), in 18 communities each; (2) conduct a robust process evaluation informed by the Consolidated Framework for Implementation Research (CFIR) [28] to explore implementation barriers and facilitators; and (3) conduct a cost evaluation to accurately estimate the costs required to start up and implement the SFP and FA intervention components and to assess the cost-effectiveness of the intervention.

### Intervention Condition

Families residing in communities randomized to the intervention group will be connected to clinically trained, trauma-informed FAs who will assess families and refer them to community services. The intervention will provide wraparound supports to prevent ACEs and substance use and, critically, enable providers and community-based partners to align their services in a way that addresses the social determinants of health (SDOH) and other community-level factors that affect substance use and the relationship between social connection and ACEs. The FA component of the intervention will run concurrently with the SFP7–17 sessions, with the FAs interacting weekly with families over the 10- to 14-week intervention period. Each week, FAs will conduct 1-hour, post-session check-ins with each family. This 1-hour period will consist of a 20-minute telephone call with families to discuss their needs, with the remaining 40 minutes used to debrief, make service referrals, and complete documentation.

### Intervention Development

The intervention development process was guided by the Problem, Objective, Design, (end-)Users, Co-creators, Evaluation, Scalability (PRODUCES) cocreation framework [29], operationalized in conjunction with the established principles of community-engaged research. PRODUCES offers a systems-oriented schema for structuring population health intervention research, emphasizing iterative coproduction of knowledge and reproducibility across all phases of inquiry—planning, implementation, evaluation, and dissemination. This framework delineates seven interdependent domains to be systematically addressed, such as (1) articulation of the public health problem or behavioral determinant targeted by the intervention; (2) specification of the overarching objectives and proximal aims of the participatory design process; (3) selection of participatory and design methodologies for cocreation; (4) identification of the intended end users and beneficiaries of the intervention; (5) characterization of the stakeholder groups engaged as codesigners; (6) determination of evaluation criteria, including fidelity, feasibility, and effectiveness metrics; and (7) specification of strategies for scalability and population-level translation.

Within this structure, we employed a co-design methodology to integrate the FA component into the evidence-based SFP. Given the existence of multiple SFP curricula with variable dosages, content emphases, and delivery modalities, we conducted final curriculum selection through an iterative, evidence-informed process that triangulated input from local implementers, assessments of organizational capacity, and feasibility analyses of delivery platforms (eg, virtual or in-person implementation). This selection process was anchored in both implementation science principles (eg, alignment with CFIR constructs) and pragmatic trial readiness (ie, feasibility of embedding the intervention in diverse real-world service systems).

The FA component itself was refined through a series of structured, iterative design sprints and stakeholder consultations with agency personnel, community representatives, and potential end users. These engagements used consensus-building techniques to ensure that the resulting intervention achieved cultural consonance with community norms and practices while maintaining fidelity to core evidence-based elements of SFP. The process also incorporated explicit attention to implementation outcomes (eg, acceptability, appropriateness, and sustainability) to strengthen downstream evaluability and translational potential. Taken together, this approach produced an intervention that is methodologically rigorous, contextually tailored, and positioned for scalable implementation, consistent with an emphasis on reproducibility, stakeholder relevance, and impact at the population level.

### Control Condition

Families residing in communities randomized to the control group will participate in the SFP7–17 group class curriculum. Parents and children participate in SFP7–17, both separately and together, as the curriculum has lessons for parents, teens, and children, plus a joint family practice class. SFP7–17 meetings are 2 hours long and are typically held in person (but families can participate remotely during extenuating circumstances). Families complete 11 sessions over a 10- to 14-week period.

### Evaluation Design

#### Overview

The study team will evaluate the intervention’s effectiveness using a modified version of a hybrid type 1 design, which is used to test a clinical intervention while gathering information on its delivery during the effectiveness trial and on the potential for implementation in real-world settings [30]. Hybrid type 1 designs are ideally situated for rigorous evaluations where there is a strong evidence base for an intervention (such as SFP) and supporting evidence that the intervention would support applicability to new delivery methods (eg, FAs) and new settings and populations (eg, communities with high rates of overdose and ACEs). Our modified hybrid type 1 design integrates process and outcome evaluation components with a cost evaluation component to evaluate the intervention’s impacts on family outcomes (eg, substance use prevalence, risk or harm perceptions, and ACEs) over a 3-year period.

**Figure 1.**
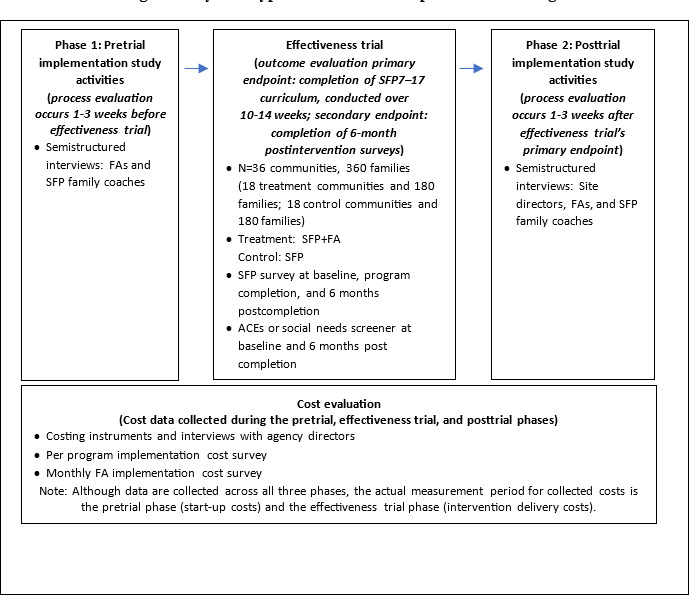
Hybrid type 1 effectiveness-implementation design. ACE: adverse childhood experience; FA: family advocate; SFP: Strengthening Families Program; SFP+FA: Strengthening Families Program and family advocate (ie, treatment condition).

Participants in each of the 36 communities (18 treatment and 18 control communities) will complete the study-related tasks for the effectiveness trial, process, and cost evaluation components over approximately 9‐11 months. Figure 1 illustrates how each evaluation component fits within the study and the associated time frames. The process evaluation’s pre- and posttrial study activities will occur 1‐3 weeks before and after the effectiveness trial, respectively. Completion of the SFP7–17 curriculum over 10‐14 weeks will be the primary end point for the effectiveness trial, and completion of 6-month postintervention surveys will be the secondary end point. The cost evaluation study activities will be conducted throughout the pretrial, effectiveness trial, and posttrial phases and reported during the posttrial component. The effectiveness trial, process, and cost evaluation components are discussed in greater detail in subsequent sections, followed by the study’s data analysis plan.

#### Effectiveness Trial

The effectiveness trial uses a cluster RCT design to randomize 36 New Jersey communities experiencing a disproportionate burden of substance use and ACEs [31] to treatment and control conditions. Cluster RCTs offer a rigorous design for evaluating community-level interventions and provide key benefits in the form of mitigating spillover effects, reducing attrition, and increasing compliance from program staff and participants [32].

#### Community Selection

All families participating in the study will come from preidentified communities across the state of New Jersey. A “community” is akin to a classified municipality in the state. New Jersey has 564 municipalities, comprising 5 location types—252 boroughs, 52 cities, 15 towns, 241 townships, and 4 villages. Communities with population sizes less than 10,000 per US Census estimates were removed from eligibility on the front end, leaving approximately 200 communities for the selection process. Eligible communities were then ranked using data comprising ACEs (collectively, these secondary data sources provided data on the prevalence of 8 of the 10 original ACEs [24], that is, physical abuse, emotional abuse, sexual abuse, physical neglect, emotional neglect, substance misuse, mental illness, and incarcerated relatives) and opioid-related vulnerability metrics, such as overdose-focused SDOH, opioid overdose–involved hospitalizations, and child maltreatment case rates [33-35].

These metrics were derived from the following secondary data sources:

- The RTI Rarity project uses supervised machine learning, including random forests and other state-of-the-art predictive methods, to create local social inequity scores at the census-tract level that draw on SDOH and other contextual, neighborhood-level measures. This study used RTI Rarity’s Local Index in drug overdose model, which provides percentile-ranked scores for each US Census tract, with higher scores indicating higher predicted drug overdose mortality rates. The model comprises 40 measures and explains 99% of the variance in drug overdose mortality rates across census tracts [36].
- Healthcare Cost and Utilization Project (HCUP) databases bring together the data collection efforts of state data organizations, hospital associations, private data organizations, and the federal government (HCUP partners) to create a national information resource of encounter-level health care data. HCUP includes the largest collection of longitudinal hospital care data in the United States, with all-payer, encounter-level information beginning in 1988.
- The National Child Abuse and Neglect Data System is a federally sponsored effort that annually collects and analyzes data on child abuse and neglect known to child protective services agencies in the United States.

Communities were subsequently assigned into quadrants based on average rankings across the 3 metrics to prioritize disproportionately affected communities. County providers with eligible communities were approached to ask about their interest in and ability to participate in the first year of the study. Vulnerable communities in counties that responded positively were then added to the list of communities to be randomized. The team also noted counties that would be able to participate in subsequent years.

#### Randomization

For the purpose of random assignment, during the 3 years of the effectiveness trial, the study team plans to enroll 12 communities from 6 counties from the initial list of counties. Each county will contribute 1 community implementing SFP-only (ie, control condition) and 1 community implementing SFP+FA (ie, treatment condition).

Counties with multiple communities will be preferred to facilitate randomization at the county level, thus helping to balance the geographical and background characteristics of the participating communities. As a contingency plan, if a county has only 1 community (or more than 1 community but with limited capacity), the study team will attempt to find matching communities in other counties. Simultaneous implementation of SFP and SFP+FA within selected counties will be preferred but is not required for successful implementation; asynchronous starts may be used in counties, if necessary.

In total, 3 general randomization approaches were identified. Feasibility and preference for a method varied by implementation and community readiness factors, as well as methodological concerns, such as control of potential confounds. The three potential approaches were pairwise assignment, pool randomization, and rolling assignment. First, under pairwise assignment, (1) communities within each county are ranked sequentially according to their ACEs, substance use disorder, and SDOH risk profile or rank; (2) sequential pairs are then randomized to SFP and SFP+FA conditions; (3) assignment is repeated for each pair; and (4) recruitment is done at the pair level. Second, under pool randomization, (1) within each county, each community is randomly assigned to 1 of 2 or more blocks or groups of communities. Block size will depend on the total number of communities available in the county. Multiple patterns of assignment of communities within each block are generated. Assignment is determined by a randomly selected pattern for each block. (2) Recruitment is then done in each county for a single SFP-only and single SFP+FA community for entry into the study. Finally, under rolling assignment, (1) assignments are based on enrollment flow and not a priori assignment; (2) recruitment takes place before assignment; and (3) the first community enrolled is randomly assigned to either SFP or SFP+FA, and the next enrolled group is automatically placed in the group not assigned to the first community.

On the basis of conversations with the study team’s New Jersey partners and information gathered from local partners, randomization was conducted in a process that combined the pairwise and pool randomization approaches. First, 2 communities were chosen at random from each county. Next, multiple blocks were generated for each county. Then, 10 blocks were generated for each county; in each block, 1 community was randomly assigned to SFP and the other was assigned to SFP+FA. The block containing the community assignment to be used was then chosen at random. The study team will repeat this process in each successive year of the effectiveness trial, for 3 rounds of randomization involving 36 communities.

#### Sample

The study team conducted power analyses to determine the minimum number of families needed to detect statistically significant effects in the effectiveness trial. Calculations were based on the use of a 3-time-point latent growth model (LGM) with linear slope conditional on treatment group (ie, a difference-in-differences model). Power analyses used a Type I error rate of 0.05 and an intraclass correlation of 0.01 to 0.05. The estimated minimal detectable effect size (based on power=0.80) for 36 communities with an assumed 10 families per site (for a total of 360 families) is 0.40 or below.

#### Eligibility Criteria

To be eligible to participate in this study, a family must meet all of the following criteria:

- Either reside or attend SFP meetings in 1 of the 36 New Jersey communities assigned to treatment or control conditions
- Consist of 1 or more adult caregivers and 1 or more children aged 7‐17 years
- Provide informed consent (caregivers) or assent (youth) to participate in the study
- Express a willingness to adhere to the regimens of the SFP and FA interventions
- Have access to necessary resources (ie, computer, smartphone, and internet access) for completing data collection instruments.

#### Objectives and End Points

Table 1 describes the study objectives and end points for the effectiveness trial. The trial will use a combination of assessments at baseline, completion, and 6-month follow-up to examine youth and parent or caregiver substance use and ACEs among families in the randomized communities. The ACEs or social needs screener will be used for both screening and data collection purposes. To mitigate potential ethical and privacy concerns, the screener does not gather separate information on types of ACEs experienced; rather, respondents are asked to indicate the number of relevant ACEs in each of 3 categories—abuse, neglect, and household challenges. Participating families will complete a baseline screener during SFP session 3, and families in the treatment group may elect to share their data with the FAs, who will assess the data and refer families to relevant services in the community. Families in the control group will be subject to a treatment-as-usual condition. The ACEs or social needs screener will be readministered 6 months post intervention to measure the intervention’s impact on the prevalence of ACEs and social needs among participating families. The study team will use weekly family referral summaries to collect outcome data to measure the intervention’s impact on clinical and nonclinical referrals.

**Table 1.** Effectiveness trial: study objectives, end points, and justifications.

Objectives	End points	Justification for end points
To use a cluster-randomized controlled trial to test the comparative effectiveness of the intervention and control condition on substance use, overdose, and ACEs^a^ in 18 communities each	Primary end points SFP^b^ posttest survey (completed by families at SFP graduation) Weekly family referral summaries (completed by SFP Family Coaches and FAs^c^ throughout intervention until SFP^b^ graduation) Community service provider follow-up survey (completed by SFP family coaches, FAs, and community service providers after SFP graduation) Secondary end points ACEs or social needs screener (completed by families at session 3 of SFP and 6 months after SFP graduation) SFP 6-month follow-up survey (completed by families 6 months after SFP graduation)	Primary end points The SFP posttest survey will collect key outcome data for measuring the intervention’s impact on substance use, perceptions of harm, and risk The weekly family referral summaries will collect key outcome data for measuring the intervention’s impact on referrals to substance use disorder services and nonclinical community service providers The community service provider follow-up survey will collect key outcome data for measuring the intervention’s long-term impact on community-level change via change in system linkages Secondary end points The ACEs or social needs screener will collect key outcome data for measuring the intervention’s impact on the prevalence of ACEs and social needs at baseline and 6 months postintervention among participating families The SFP 6-month follow-up survey will collect key outcome data for measuring the intervention’s long-term impact on substance use, perceptions of harm, and risk

a ACE: adverse childhood experience.

b SFP: Strengthening Families Program.

c FA: family advocate.

To assess community-level impact, the trial will use a survey of community service providers to collect data on system-level changes in the referral networks among FAs, SFP facilitators, and community service providers in the 36 participating communities. The survey will ask respondents about the characteristics of their respective organizations (eg, number of clients served and staff size) and services offered to individuals. Via a series of network questions, respondents will be asked to list up to 10 of the main organizations that their organization refers to or collaborates with. Finally, respondents will be asked whether their organization refers to or collaborates with specific community service providers. Responses to these surveys will provide a matrix of interactions among FAs, SFP facilitators, and community service providers that will help construct egocentric clinical and nonclinical referral networks within each of the 36 communities for the periods before and after intervention implementation. The analysis methods for the effectiveness trial and other evaluation components are discussed in the Data Analysis section of this paper.

### Process Evaluation

The process evaluation will consist of pretrial implementation data collection, fidelity monitoring during intervention implementation, and posttrial implementation data collection. Pre- and posttrial process evaluation data collection activities were informed by the CFIR to allow the study team to explore implementation barriers and facilitators by identifying strategies that facilitate the integration of the FA role into SFP. The process evaluation aims to help clarify (1) the barriers and facilitators that families in participating communities may encounter in accessing services; (2) the challenges that participating agencies face in implementing SFP and integrating FA into SFP, as well as any solutions; and (3) the ways in which the implementation of SFP+FA affects family access to services and cross-system coordination and communication among agencies. Table 2 describes the study objectives and end points for the process evaluation.

**Table 2.** Process evaluation: study objectives, end points, and justifications.

Objectives	Primary end points	Justification for primary end points
To conduct a robust process evaluation informed by the Consolidated Framework for Implementation Research in order to explore implementation barriers and facilitators	Posttrial interviews (completed with SFP^a^ and FA^b^ delivery staff after SFP^a^ graduation) Posttrial program lead interviews (completed with prevention agency program leads after SFP graduation)	Both types of posttrial interviews will collect key process evaluation data related to observed challenges and associated solutions that participating agencies encountered when integrating the FA component into SFP observed barriers and facilitators that families encountered when accessing services ways in which the implementation of the intervention affected family access to services, cross-system coordination, and communication

a SFP: Strengthening Families Program.

b FA: family advocate.

During the pretrial implementation phase, the study team will conduct 2 series of qualitative interviews. The first series will interview designated SFP facilitators from each participating community. These semistructured interviews will gather qualitative data about SFP facilitators’ past experiences implementing the SFP, their understanding of the SFP facilitator’s role in the program, anticipated challenges of implementing the program, and anticipated barriers to and facilitators of accessing services that may be encountered by families in their communities. The second series of interviews will be conducted with FAs, with each FA participating in 1 pretrial interview. These semistructured interviews will gather qualitative data regarding FAs’ understanding of their role and any anticipated challenges and facilitators pertaining to their role.

During intervention implementation, the study team will monitor implementation fidelity through weekly fidelity reporting. Each site will have 1 designated SFP facilitator and parents or caregivers who will respond to brief fidelity questionnaires after each SFP session. SFP facilitators will report session attendance, adherence to the curricula, and participant engagement, whereas parents and caregivers will report on their personal satisfaction with the session and their perceptions of SFP facilitators’ knowledge, empathy, and preparedness.

During the posttrial process evaluation, the study team will conduct 3 series of qualitative interviews. The first series of semistructured interviews will gather qualitative data about facilitators’ experiences implementing SFP and about barriers to and facilitators of accessing services encountered by families in their communities. These interviews will also ask SFP facilitators from intervention communities about their experiences with the addition of the FAs. The second series will interview FAs and will gather qualitative data regarding FAs’ experiences in their role, including any challenges and facilitators they encountered. The third and final series will interview prevention agency program leads who oversee SFP groups to gather qualitative data regarding each agency’s experience implementing SFP and regarding barriers to and facilitators of accessing services encountered by families in their communities. The study team will also ask program leads from intervention communities about their experiences with the integration of the FA role.

### Cost Evaluation

As illustrated in Table 3, the cost evaluation will use data collected from prevention agency program leads, SFP facilitators, and FAs to identify the activities and costs associated with starting up and implementing the intervention in treatment and control communities. The data collected from prevention agency program leads will include cost data on staff salaries, overhead costs, training materials and fees, and other SFP implementation resources to inform accurate cost estimates for prespecified activities (eg, management); these data will be supplemented by survey data collected from SFP facilitators and FAs. Given that SFP facilitators and FAs are the main staff delivering the intervention, their survey data will help to understand how their time is spent in various activities, including travel time and distance for SFP sessions, time spent in various activities related to implementing SFP, and time spent in specific FA activities (eg, engaging with families). The study team will use the activity-based data collected from SFP facilitators and FAs, combined with the cost information from the prevention agency program leads, to calculate the costs of the treatment and control conditions and to examine potential sources of variation in costs, such as activity cost differences or staffing mix differences. Start-up costs for the intervention will also be estimated from preimplementation activity data, primarily from the cost instrument completed by prevention agency program leads.

**Table 3.** Cost evaluation: study objectives, end points, and justifications.

Objectives	Primary end points	Justification for primary end points
To conduct a cost evaluation to accurately estimate the costs required to implement SFP^a^+FA^b^ intervention components and assess the comparative cost-effectiveness of the treatment intervention and control To estimate the start-up costs associated with the intervention	Costing instrument and interview (completed by prevention agency program leads after SFP graduation) Per-program implementation cost survey (completed by SFP^a^ Family Coaches after SFP graduation) Monthly implementation cost survey (completed by FAs monthly)	The costing instrument and interview, per program implementation cost survey, and monthly implementation cost survey will collect key cost evaluation data to inform start-up cost estimations, implementation cost estimations, and cost-effectiveness analyses

a SFP: Strengthening Families Program.

b FA: family advocate.

### Data Analysis

**Figure 2.**
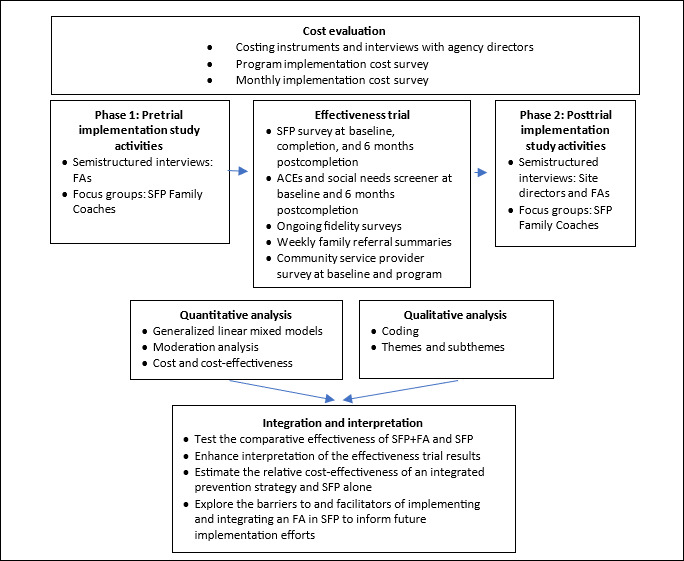
Overview of the mixed methods analysis for the study’s hybrid type 1 design. ACE: adverse childhood experience; FA: family advocate; SFP: Strengthening Families Program.

Figure 2 details the mixed methods analysis approach that will be used to analyze data from the evaluation’s process, effectiveness trial, and cost components. In the sections below, we provide an overview of the methods used for each component, followed by the approach used to systematically integrate and analyze the findings across components.

### Quantitative Analysis of Effectiveness Trial Data

#### Overview

All inferential statistics will use a Type I error rate of 0.05 and 2-tailed tests. The study team will apply a multiple comparison adjustment to mitigate the likelihood of Type 1 errors across the analyses of effectiveness trial data. The Benjamini-Hochberg procedure (Benjamini and Hochberg [37]) will be applied separately for each set of outcomes within the primary and secondary end points specified in Table 1 to adjust *P* values and account for multiple hypothesis testing.

#### Descriptive Statistics

Preliminary analyses will be used to characterize and explore the data. Means, frequencies, and correlations will describe the distributions of demographics, key covariates, and the outcome variables. Simple bivariate regression models will be used to examine the groups for baseline differences. Variables will be assessed for outliers and other distributional violations.

#### Program Effects

The difference in family outcomes between exposure to SFP+FA and SFP-only will be examined with generalized linear mixed models (GLMMs)—that is, hierarchical linear models or multilevel regression models. These models can accommodate the repeated assessments at baseline, immediate post treatment, and 6-month follow-up. GLMM can simultaneously account for nesting of families in communities to account for any nonheterogeneity attributable to location. The evaluation model will consist of a 3-time-point LGM estimated within the GLMM framework. This model examines how family outcomes (eg, substance use prevalence, risk or harm perceptions, and ACEs) change over time and incorporates random effects (variance components) for each family’s initial status (intercept) and rate of change or slope [38]. The LGM model will test how the SFP and SFP+FA groups change from baseline through the 6-month follow-up and the difference in those 2 rates of change. The difference in rates of change (sometimes called a difference-in-differences model) [39,40] will be the primary evaluation outcome.

**Figure 3.**
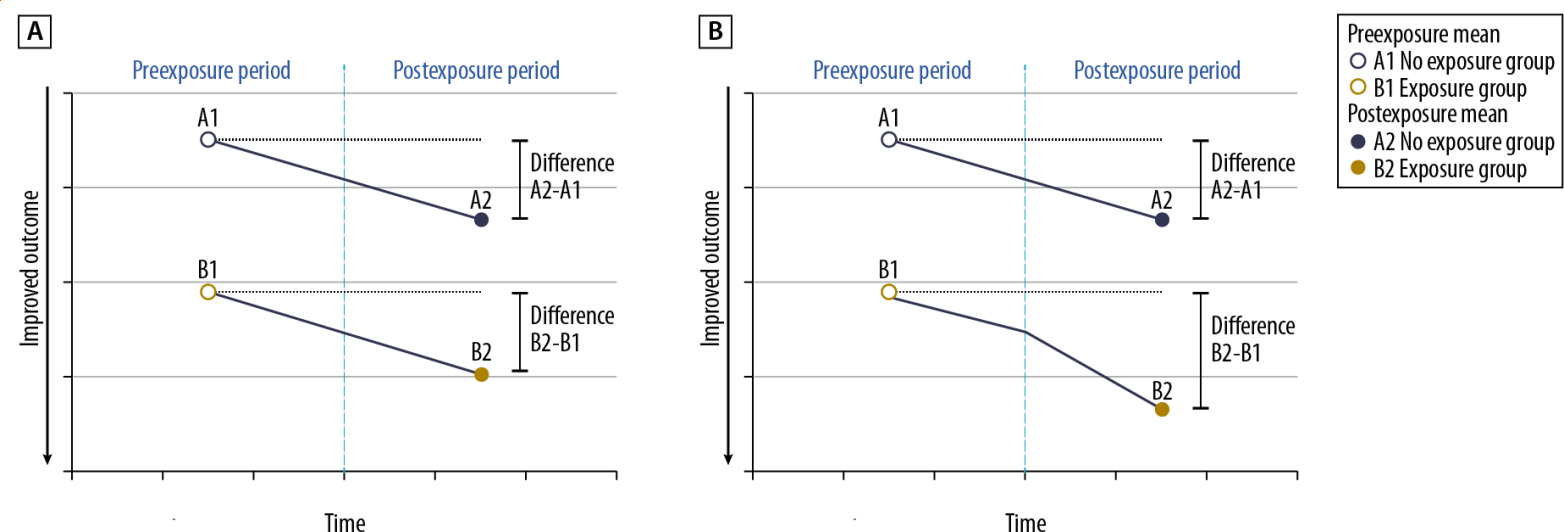
Illustration of difference-in-differences evaluation model. (A) No association between exposure and measured outcome, and (B) association of exposure and measured outcome.

Figure 3 illustrates a difference-in-difference model in which panel A shows no intervention effect (the difference A2−A1 is the same as the difference B2−B1), and panel B demonstrates an intervention effect (A2−A1 is not equal to B2−B1).

The LGM approach provides more options than ANOVA or other traditional mean comparison techniques by being able to incorporate covariates into an analysis. Covariates may be at any level included in the GLMM, such as the individual, family, or community levels. All outcomes assessed will be examined with these models and will begin with random effects at the family and community levels for both the intercept and slope, and the covariance between intercepts and slopes at each level will be included. Models may be iteratively adjusted to remove random effects and covariances as necessary, because it is common for not all outcomes of interest to require all sources of variability.

#### Missing Data Strategies

The impact of missing data will be minimized through the use of likelihood-based estimators, which are available for multilevel models of the proposed difference-in-differences model for the evaluation. These likelihood methods yield unbiased estimates and maximally efficient standard errors without sacrificing cases (thus maximizing statistical power). The proposed method of accommodating missing data is fully appropriate when data are missing truly at random (missing completely at random [MCAR]) or predicted by other variables (such as baseline scores and covariates) in a given model but independent of the potential values of the outcome itself (ie, missing at random [MAR]). The MAR and MCAR mechanisms are termed ignorable missing data because they can be rendered unproblematic for analysis with the proper estimation technique and model (eg, including the variable that predicts missingness as a predictor of the outcome). Although there is no general test of the MAR assumption, MAR-appropriate estimators are typically maximally efficient and unbiased even when the data are not truly MAR or MCAR (ie, the missing data are nonignorable). However, if the pattern of missing data is suspected to be nonignorable, then the research team will incorporate auxiliary variables in the analysis model or use pattern-mixture models that are adept at obtaining estimates for longitudinal studies with attrition caused by levels of outcomes.

### Network Analysis of Effectiveness Trial Data

The findings from the community service provider survey will be used to examine the intervention’s effectiveness in achieving community-level change via change in system linkages within the referral networks between FAs, SFP family coaches, and community service providers in each of the 36 communities participating in the effectiveness trial. Surveys will be administered pre- and postimplementation of the intervention in each of the 36 communities, with individuals in the 3 respondent categories asked a series of questions about the organizations they refer to or collaborate with for any ACEs, substance use, or mental health services. Respondents (referred to as egos in the community service provider surveys) will be asked to list up to 10 of the main organizations that they refer or collaborate with and then to respond to a second set of questions asking about referrals and collaborations with selected community service providers. Answers to these questions will provide a matrix of interactions among FAs, SFP family coaches, and community service providers that will facilitate the construction of egocentric networks within each of the 36 communities for the periods before and after implementation of the intervention.

This study will use a multistep process to analyze the networks for the treatment and control communities. In the first step, network diagrams will be constructed using the R software package (R Core Team). Descriptive network analyses will be conducted on each of the networks to examine key network measures for the communities in the treatment and control conditions. These analyses will move from the simple to more complex network measures:

- Network size: The number of nodes (ie, FAs, SFP family coaches, and community service providers) in a given network.
- Edges: Connections (directed and undirected) among nodes within a network.
- Density: How close a network is to being fully connected, ranging from no connections at all to all possible connections among nodes being made.
- Centrality: The degree to which a network is organized around a central node by comparing the proportion of edges to a central node to all other edges in a network.
- Reciprocity: The fraction of all possible connections in which nodes are mutually connected.
- Triadic closure: The addition of an edge that closes a 2-path (ie, 3 nodes connected by 2 edges) to form a triangle (or among 3 nodes, A, B, and C, connections A–B and A–C exist, and a new connection B–C is formed).
- Assortativity: The extent to which nodes in a network are associated with other nodes in the network, being of similar sort or being of opposing sort.

In the next step of the analysis, mixed effects models will be estimated to examine the intervention’s effectiveness in achieving community-level change via overall and configurational changes in the referrals and linkages between FAs, SFP family coaches, and community service providers in treatment and control communities. The random intercept and random coefficient mixed effects models will incorporate a 2-level structure with repeated measurements (ie, pre- and postimplementation) nested within communities [41]. The model will incorporate random slopes for communities and random intercepts for time. The following 2-level model will estimate the impacts of the intervention on referrals and linkages between FAs, SFP family coaches, and community service providers:


$$y_{ij}=\;\beta_{0}+\;\mu_{0j}+\beta_{1}Treat_{1\;ij}+\beta_{2}X_{2\;ij}+\ldots +\beta_{p}X_{p\;ij}+\;\mu_{1}X_{1ij}+\varepsilon_{ij}$$

where $y_{ij}$ refers to the outcome for time point i in community j, $\beta_{0}$ is the overall mean of the outcome across all groups, $\mu_{0j}$represents the random intercept, $\beta_{1}{Treat}_{1ij}$ is an indicator equal to 1 for communities assigned to the treatment group and zero for those assigned to the control group, $\beta_{2}X_{2\;ij}+\ldots +\beta_{p}X_{p\;ij}$ represents a vector of covariates, $\mu_{1}X_{1ij}$ represents the random coefficient, and $\varepsilon_{ij}$ is an individual-level error term.

The final step of the analysis will consist of formulating exponential random graph models [42] (ERGMs), a class of statistical models for social networks, to examine the structure of networks in the treatment and control communities post implementation. ERGMs account for the presence and absence of network ties (ie, edges) and provide an approach to modeling overall network structure by modeling small, local, tie-based structures, such as reciprocity and triangles. These models permit inferences about whether a given network has significantly more or less of a given feature (such as reciprocity or triadic closures) than one would expect and provide a deeper understanding of how and why network ties arise. This study will estimate a series of ERGMs to model network features in the treatment and control group communities [42]:


$$P_{\theta \;}\left(G\right)=ce^{\theta 1z1\;\left(G\right)+\theta 2z2\;\left(G\right)+\ldots +\;\theta pzp\;\left(G\right)}$$

where the probability of a given network *G* is given by a sum of network statistics (z in the equation above, which represents counts of the number of network configurations in the given network *G*) weighted by parameters (θ in the equation above) inside an exponential (and where c is a normalizing constant).

### Qualitative Analysis of Process Evaluation Data

The study team will analyze qualitative data using a hybrid deductive-inductive analytic approach that emphasizes addressing key constructs of the CFIR, such as intervention characteristics, outer and inner settings, characteristics of individuals, and process, while allowing for emerging patterns. Interview transcripts will be uploaded to the qualitative data analysis software ATLAS.ti (Lumivero). Individual team members will code interview transcripts using a codebook that is guided by CFIR domains and the constructs within them, and that allows team members to identify emerging themes in the data. Furthermore, 2 coders (KJ and FW) will code a subset of transcripts and compare the level of agreement to assess reliability. The study team will calculate the Cohen κ statistic to check for coder consistency in applying the codebook. If a high level of consensus is not reached, team members will work to resolve conflicts until reaching a level of high intercoder agreement (0.80).

Through an iterative process, the qualitative study team will continually create, refine, and discard codes. The qualitative study team will hold twice-weekly data meetings during the analysis phase to discuss coding. As transcripts are discussed and coded, the study team will also write analytical memos to record observations, refine preliminary themes, and capture emerging themes. When all transcripts have been coded and discussed, the study team will engage in an iterative process of searching data using ATLAS.ti, comparing data segments from searches, and writing analytic memos to answer the process evaluation’s research questions, thereby developing a full understanding of the barriers to and facilitators of implementing FAs within SFP.

### Quantitative Analysis of Cost Evaluation Data

The study team will use the collected cost data to calculate average program start-up costs and ongoing program implementation costs, primarily from the funder perspective but also from the societal perspective, reflecting the costs from the perspectives of all funders, plus the value of any in-kind donations of time or other supplies or equipment. In cost analyses, costs will be calculated for the following resource categories: labor, materials or supplies, equipment, and other resource categories. Mean costs will be calculated by intervention assignment (SFP+FA or SFP-only), and cost variability will be examined by intervention, staff mix, or communities. The sensitivity of costs will also be examined against assumptions about unit costs or other input values. Cost estimates, particularly the costs of SFP, will be compared with other estimates in the research literature, along with the primary drivers of differences or similarities in costs across implementations of SFP. These costs will be reported as costs per site per year and costs per family per year.

The total program costs for SFP+FA and SFP will be estimated and subsequently used to calculate the incremental cost of SFP+FA relative to SFP-only. These analyses will compare differences in start-up costs and ongoing program costs to implement the programs during the study period. Fixed costs, which do not vary based on the number of families served, will be compared with variable costs, which increase for each family or site added. Understanding the breakdown of costs between start-up and implementation is necessary for accurately estimating the costs of program scale-up or new program implementation in other settings.

The study team will also explore the feasibility of conducting a budget impact analysis. A budget impact analysis would first identify the organizations and agencies that incur costs and experience cost savings resulting from SFP+FA or SFP-only. These interventions may affect service usage in multiple sectors, including the health care system, the criminal justice system, and the child welfare or protective services system. A budget impact analysis will consider a time horizon beyond the trial period, such as 5 or 10 years, to analyze whether and how the intervention affects longer-term outcomes in each of the various systems that affect families. One aspect of this feasibility study is to determine whether data are available to estimate the budget impact from the state, federal, or other funder perspectives. A potential barrier is limited access to appropriate data from all relevant systems to conduct the analysis. Although it is possible to assess impacts on emergency department and inpatient health care usage using local data from Medicaid claims or hospital discharges, it may be more challenging to obtain data from nonhealth social services systems. An important part of this study will be to assess the feasibility of obtaining relevant data from nonhealth social services systems.

### Mixed Methods Analysis of Process, Effectiveness, and Cost Data

In the final phase of the analysis, the evaluation data will be analyzed using a mixed methods analysis approach in which qualitative and quantitative data collected from the process, outcomes, and cost components of the hybrid type 1 design are systematically integrated and analyzed. Mixed methods analysis helps answer questions that could not be answered by the qualitative or quantitative approaches alone, thereby enriching results, strengthening the overall reliability of a study’s findings, and providing the opportunity to uncover unique insights and findings that would have been otherwise neglected using a single method. The study’s mixed methods analysis provides a systematic approach to integrating and interpreting data from the outcome evaluation’s effectiveness trial, the process evaluation’s focus on implementation barriers and facilitators, and the cost evaluation’s cost and cost-effectiveness estimates.

The findings from the qualitative and quantitative analyses of process, outcome, and cost data will be systematically integrated using joint display tables that provide a visual framework for intentionally integrating findings with a clear rationale, thereby illuminating insights beyond separate quantitative and qualitative analyses. This mixed methods analysis provides a critical benefit to the study by providing a robust understanding of the intervention’s effectiveness, implementation barriers and facilitators, and cost-effectiveness, and thereby enhancing the likelihood that the intervention strategies shown to be effective will be sustainable when implemented in future communities.

### Ethical Considerations

The study protocol, data collection instruments, and consent forms were approved by the Advarra Institutional Review Board (#00071669). The study is conducted in accordance with the ethical principles of the Belmont Report, with the study’s effectiveness trial conducted in accordance with International Council on Harmonisation Good Clinical Practice, applicable United States Code of Federal Regulations, and the NCIPC Terms and Conditions of Award. Important protocol modifications will be shared with the institutional review board, trial registries, journals, and trial participants.

Informed consent will be obtained from all participating parents and caregivers, and parental permission and assent will be obtained for all participating youth. Consent and assent procedures will be conducted electronically and designed to ensure participants’ understanding of study procedures and the associated risks and benefits of participation. Participants will be compensated for their time using electronic gift cards for engaging with intervention activities and completing surveys or interviews. All consent forms, surveys, and interview data are stored within a Health Insurance Portability and Accountability Act (HIPAA)–compliant network that is accessible only to authorized project staff using password-protected devices. No identifiable information will appear in reports or publications.

## Results

Funding was awarded in 2022, and institutional review board approval was obtained in November 2023. Intervention and evaluation development lasted approximately 1 year, with study partners engaged in the co-design process to ensure alignment with the principles of community-placed behavioral health research. Study recruitment began in November 2023 and is anticipated to continue until September 2026. Final data collection is anticipated by March 2027, followed by data analysis.

## Discussion

### Strengths and Limitations

One of the key strengths of this study is the use of a hybrid type 1 effectiveness-implementation design, which allows for the simultaneous evaluation of both the clinical outcomes and the implementation processes in real-world settings. This comprehensive approach provides valuable insights into not only whether the intervention works, but also how it can be implemented effectively in community settings. The cluster RCT design involving 36 New Jersey communities further strengthens the study by reducing the likelihood of selection bias and enabling the comparison of intervention and control groups at the community level. The study’s integration of both family- and community-level interventions addresses multiple SDOH and enhances the potential for sustainable, long-term impact on reducing ACEs and substance use.

However, the study has certain limitations that could affect the interpretation of its results. First, measuring compliance with family referrals and tracking the use of community resources may be challenging, potentially leading to incomplete data. Furthermore, the aggregation of family-level outcomes to assess community-level impact could introduce variability that might obscure the intervention’s true effect. The reliance on self-reported data, particularly regarding sensitive topics such as ACEs and substance use, could also introduce response bias. Finally, the study’s focus on communities in New Jersey limits the generalizability of its findings to other regions with different demographic and socioeconomic characteristics.

### Implications for Practice

The findings of this study have important implications for both policy and practice, particularly in addressing the dual challenges of substance use and ACEs through community-based interventions. If successful, the integration of trauma-informed FAs with the SFP could serve as a scalable model for addressing ACEs and substance use prevention in other high-risk communities. By aligning services and resources across family and community levels, this approach could inform the development of more comprehensive, multilevel interventions that are responsive to the specific needs of families and communities. Additionally, the study’s process evaluation will provide valuable insights into the barriers to and facilitators of implementing such interventions, helping practitioners and policymakers identify strategies to enhance service delivery and improve family outcomes.

### Conclusions

This study addresses a key gap in the evidence base by rigorously evaluating a novel intervention that effectively integrates family- and community-level factors to prevent substance use, overdose, and ACEs. The study’s research design will build the evidence base for community-level interventions by using a hybrid type 1 design that integrates outcome, process, and cost evaluations to optimize the likelihood that the intervention strategies shown to be effective will be sustainable when implemented in future communities. The findings from this study will provide a roadmap for how disproportionately affected communities can implement the intervention to prevent substance use, overdose, and ACEs.

## Abbreviations

ACE: adverse childhood experience
CDC: Centers for Disease Control and Prevention
CFIR: Consolidated Framework for Implementation Research
ERGM: exponential random graph models
FA: family advocate
GLMM: generalized linear mixed model
HCUP: Healthcare Cost and Utilization Project
HIPAA: Health and Insurance Portability and Accountability Act
LGM: latent growth model
MAR: missing at random
MCAR: missing completely at random
NCIPC: National Center for Injury Prevention and Control
PRODUCES: Problem, Objective, Design, (end-)Users, Co-creators, Evaluation, Scalability
RCT: randomized controlled trial
RTI: Research Triangle Institute
SDOH: social determinants of health
SFP: Strengthening Families Program
